# The PARP inhibitor AZD2281 (Olaparib) induces autophagy/mitophagy in *BRCA1* and *BRCA2* mutant breast cancer cells

**DOI:** 10.3892/ijo.2015.3003

**Published:** 2015-05-12

**Authors:** Banu Arun, Ugur Akar, Angelica M. Gutierrez-Barrera, Gabriel N. Hortobagyi, Bulent Ozpolat

**Affiliations:** 1 Department of Breast Medical Oncology, The University of Texas M.D. Anderson Cancer Center, Houston, TX 77030, USA; 2 Department of Experimental Therapeutics, The University of Texas M.D. Anderson Cancer Center, Houston, TX 77030, USA

**Keywords:** PARP inhibitors, AZD2281, BRCA mutation, allelic loss, breast cancer, autophagy, mitophagy, therapy

## Abstract

PARP inhibitors are considered promising anti-cancer agents and currently being tested in clinical trials in hereditary breast cancer patients harboring mutations in *BRCA1* and *BRCA2* genes. In this study, we investigated the antiproliferative effects and mechanism of PARP inhibitors ABT-888 (Veliparib), BSI-201 (Iniparib) and AZD228 (Olaparib) in breast cancer cell lines with *BRCA1* or *BRCA2* mutations and 9 different BRCA wild-type cell lines with *BRCA1* allelic loss. We found that AZD2281 was the most potent in the PARP inhibitors and induces significant growth inhibition (~95%) in *BRCA1* mutant (HCC-1937, MDA-MB-436, and SUM-149PT) and *BRCA2* mutant (HCC-1428) cell lines. AZD2281 treatment also resulted in growth inhibition ranging from 20 to 50% in cells with *BRCA1* allelic loss, including ER(+), HER2/Neu(+) and triple-negative breast cancer (TNBC) cells, but showed no effect in cells without with type BRCA without allelic loss. Knocking down of *BRCA1* or *BRCA2* in TNBC cells with *BRCA1* allelic loss by RNA interference significantly enhanced AZD2281-induced growth inhibition and induced significant autophagy that was associated with mitophagy in cells with *BRCA* mutations. Inhibition of autophagy by gene knockdown significantly diminished AZD2281-induced mitophagy and apoptosis, indicating that autophagic process mediates some of the downstream effects of PARP inhibitors. In conclusion, our data provide the first evidence of PARP inhibitor AZD2281 autophagy and mitophagy in breast cancer cell lines with BRCA mutations or BRCA-allelic loss. In addition, our results indicate that the patients with *BRCA1* allelic loss may also benefit from PARP inhibitor therapy if BRCA is further inhibited.

## Introduction

Breast cancer is the most frequent malignancy in women and the second leading cause of cancer death among women in the United States (1,2). A family history of breast cancer is one of the most important risk factors for the disease (3). In addition to the two major breast cancer susceptibility genes, *BRCA1* and *BRCA2*, several other genes associated with breast cancer predisposition have been identified, including *ATM, CHEK2, PALB2, RAD51C* and *BRIP1*. Many of these genes are associated with *BRCA1* and *BRCA2* in the DNA damage response (DDR) pathway (4).

Germline mutations of *BRCA1* predispose female carriers to breast and ovarian cancers (5). Although germline mutations in *BRCA1* account for only 5% of breast cancer cases, silencing of *BRCA1* by promoter hypermethylation and other mechanisms may contribute to ≤30% of sporadic breast cancers (6,7). *BRCA1*-associated breast cancers usually contain *p53* mutations and often exhibit a triple-negative phenotype (8,9). *BRCA1* and *BRCA2* have roles in homologous recombination (HR) for DNA repair (10,11). When the remaining wild-type allele is lost in a tumor precursor cell, this repair mechanism does not work, resulting in genomic instability that is sufficient to enable tumor development (12,13). Most cancers have defects in some part of the DDR pathway. This provides an opportunity for therapeutic intervention as genotoxic therapies cause significant DNA damage, which is repairable in healthy cells but not in DDR-defective cancer cells.

PARP family of proteins (PARP1 and PARP2), are involved in a number of critical cellular processes, including DNA damage repair and programmed cell death (14). When activated by DNA damage, these proteins recruit other proteins that do the actual work of repairing DNA. Inhibition of PARP is a recently developed strategy for cancer therapy that exploits DDR defects in cancer cells (14). PARP is responsible for the sensing and repair of single-strand DNA breaks via base excision repair (15). When a replication fork encounters a single-strand break, the result is a double-strand break. In wild-type cells, these double-strand breaks are often repaired via homologous recombination (16). Cells deficient with BRCA1 and BRCA2 are unable to repair these double-strand breaks efficiently and therefore undergo cell death (17,18). Thus, PARP inhibitors exhibit efficacy in breast cancers with inherited mutations in *BRCA1* or *BRCA2* (19). PARP inhibitors, Olaparib (AZD2281), Veliparib (ABT-888), and Iniparib (BSI-201) have been shown to be promising anti-cancer agents for breast and ovarian cancer and being tested in clinical trials. Recently, the orally active PARP inhibitor AZD2281 was evaluated as a single-agent therapy in humans and showed clinical antitumor activity in BRCA-associated cancers (19,20). However, the mechanism of action of PARP inhibitors alone in cancer cells is not fully understood.

In this study, we investigated the effects of PARP inhibitors in *BRCA1* or *BRCA2* mutant breast cancer cell lines and in wild-type *BRCA* cell lines with and without BRCA1 allelic loss. We provide evidence that the PARP inhibitor AZD2281 inhibits the growth of breast cancer cells with BRCA1 allelic loss lacking mutation in *BRCA1*. These results might lead the way to new approaches for treating a broad spectrum of breast cancer subtypes. We also demonstrated that the PARP inhibitor AZD2281 induces autophagy in BRCA mutated breast cancer cells as well as breast cancer cells with BRCA1 allelic loss lacking mutation in *BRCA1*. Our results also indicate importance of selection of patients who would benefit from PARP inhibitor therapy and molecular subclassifications of BRCA-related breast cancers.

## Materials and methods

### Cell lines, culture conditions, and reagents

We studied 14 human breast cancer cell lines: 3 *BRCA1* mutant lines with *BRCA1* allelic loss (HCC-1947, MDA-MB-436, and SUM-149PT), 1 *BRCA2* mutant line with *BRCA2* allelic loss (HCC-1428), 9 BRCA wild-type lines with *BRCA1* allelic loss (MCF-7, ZR75, MDA-MB-361, BT-474, SKBR3, MDA-MB-231, BT-549, MDA-MB-468 and BT-20), and 1 BRCA wild-type line without *BRCA1* allelic loss (T47D). T47D, MCF-7, ZR75, MDA-MB-361, BT-474, SKBR3, MDA-MB-231, BT-549, MDA-MB-468, and BT-20 cells were cultured at 37°C in DMEM supplemented with 10% FBS in a humid incubator with 5% CO_2_. SUM-149PT cells were cultured in Ham’s F-12 supplemented with 5% FBS, insulin, and hydrocortisone. The PARP inhibitors veliparib (ABT-888), olaparib (AZD2281), and iniparib (BSI-201) were purchased from Selleck Chemicals (Houston TX, USA).

### WST-1 assay

Cell viability was assayed by applying the cell proliferation reagent WST-1 (Roche Applied Science). First, a suspension of 4,000 cells per 90 μl was seeded into each well of a 96-well plate and cultured overnight. Then, the necessary amount of PARP inhibitor was added to the individual wells. After 3 days of PARP inhibitor treatment, 10 μl of the ready-to-use WST-1 reagent was added directly into the medium, the plates were incubated at 37°C for 30 min, and absorbance was measured on a plate reader at 450 nm. All experiments were done in triplicate. Cell viability was calculated as the percentage of cells killed by the treatment as measured by the difference in absorbance between treated and untreated wells.

### Cell transfections

Lentiviral particles expressing BRCA1, BRCA2, ATG5, or control shRNA were purchased from Sigma. MDA-MB-231, BT-20, and HCC-1428 cells were transfected at a multiplicity of infection of 5. Five days after transfection, cells were treated with 5 μg/ml of puromycin concentration to select cells stably expressing shRNA. Lentiviral vector expressing mitochondrial yellow fluorescent protein (mYFP) was purchased from Biogenova. HCC-1428 cells were transfected at a multiplicity of infection of 5.

### Western blot analysis

After treatment, the cells were trypsinized and collected by centrifugation, and whole-cell lysates were obtained by using a cell lysis buffer. Total protein concentration was determined by using a detergent-compatible protein assay kit (Bio-Rad Laboratories). Aliquots containing 30 μg of total protein from each sample were subjected to SDS-PAGE with a 12% gradient and electrotransferred to nitrocellulose membranes. The membranes were blocked with 5% dry milk in TBS-Tween-20 and probed with primary antibodies against BRCA1 and BRCA2 (Cell Signaling Technology) and LC3 (Sigma). The antibodies were diluted in TBS-Tween-20 containing 2.5% dry milk and incubated at 4°C overnight. After the membranes were washed with TBS-Tween-20, they were incubated with horseradish peroxidase-conjugated anti-rabbit or anti-mouse secondary antibody (Amersham Life Sciences). Mouse anti-β-actin and donkey anti-mouse secondary antibodies (Sigma) were used to monitor β-actin expression to ensure equal loading of proteins. Chemiluminescence was detected with ChemiGlow detection reagents (Alpha Innotech). The blots were visualized with a FluorChem 8900 imager and quantified with densitometer software (Alpha Innotech).

### Evaluation of acidic vesicular organelles

To detect and quantify acidic vesicular organelles, cells were stained with acridine orange as described previously (21). The number of acridine orange-positive cells was determined by fluorescence-activated cell sorting (FACS) analysis.

### Transmission electron microscopy

Cells were grown on 6-well plates, treated with AZD2281, ATG5 shRNA, or control shRNA, fixed for 2 h with 2.5% glutaraldehyde in 0.1 mol/l cacodylate buffer (pH 7.4), and postfixed in 1% OsO_4_ in the same buffer and then subjected to the electron microscopic analysis as described previously. Representative areas were chosen for ultrathin sectioning and viewed with a Hitachi 7600 electron microscope (Japan).

### Flow cytometry analysis of apoptosis

Cells were collected and double-stained with Annexin V-fluorescein isothiocyanate (FITC) and propidium iodide using an Annexin V-FITC apoptosis detection kit (BD Pharmingen) and evaluated with a flow cytometer.

## Results

### AZD2281 inhibits cell survival in BRCA1 or BRCA2 mutant breast cancer cell lines

**Figure 1:**
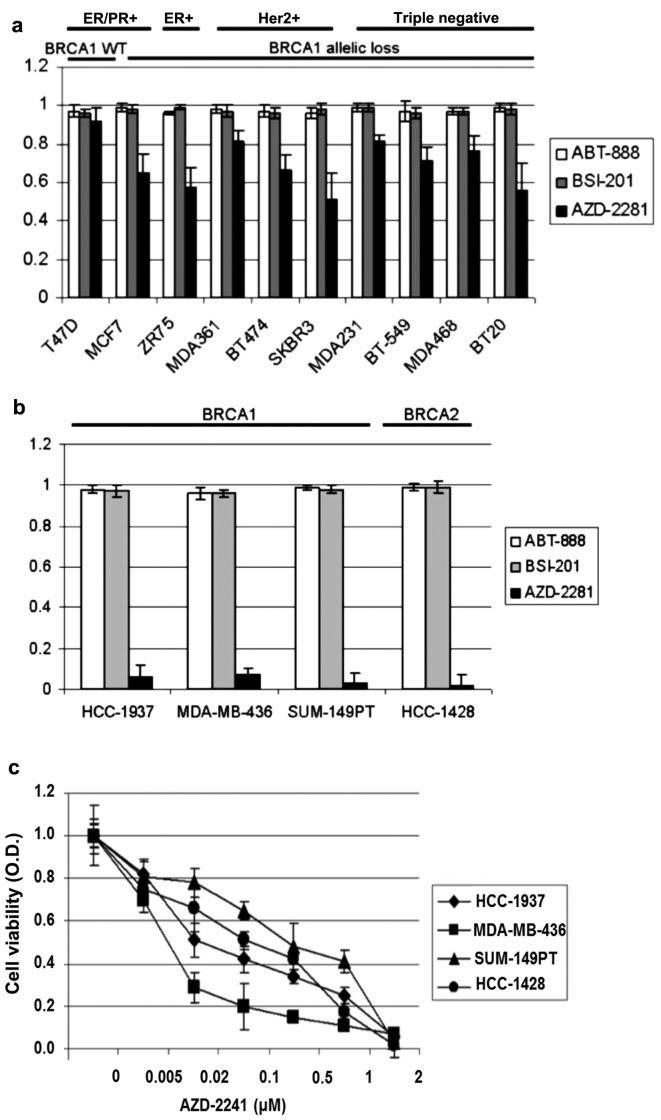
AZD2281 treatment inhibits cell survival in breast cancer cell lines with or *BRCA1* allelic loss, *BRCA1* and *BRCA2* mutations. (a) BRCA wild-type ER/PR^+^, ER^+^, HER2/Neu^+^, and triple-negative cell (TNBC) lines with *BRCA1* allelic loss were treated with 3 different PARP inhibitors at 2 μM and cell survival was evaluated at day 4. (a) *BRCA1* or *BRCA2* mutant breast cancer cell lines were treated with 3 different PARP inhibitors at 2 μM. (c) *BRCA1* or *BRCA2* mutant breast cancer cell lines were treated with different concentrations of AZD2281. NT, no treatment. All growth rates were measured with the WST-1 assay.

According to the literature, 5 (12%) of 41 breast cancer cell lines have BRCA mutations and 28 (68%) of the 41 cell lines have *BRCA1* allelic loss. To investigate the effects of PARP inhibitors in BRCA wild-type breast cancer cell lines with BRCA allelic loss we treated BRCA wild-type ER/PR^+^, ER^+^, HER2^+^, and triple-negative cell lines with 3 different PARP inhibitors, ABT-888, BSI-201, and AZD2281, for 4 days. Growth rates were measured with the WST-1 assay. Whereas AZD2281 induced an average growth inhibition of 33% in BRCA wild-type cell lines at 2 μM, ABT-888 and BSI-201 did not induce growth inhibition in the same cell lines at 2 μM concentration. The growth inhibition effect of AZD2281 was significantly higher in the BRCA wild-type cell lines with *BRCA1* allelic loss than in the BRCA wild-type cell line without *BRCA1* allelic loss (Fig. 1a). We also used the same PARP inhibitors at the same concentration (2 μM) in the *BRCA1* mutant (HCC-1937, MDA-MB-436, and SUM-149PT) and *BRCA2* mutant (HCC-1428) cell lines. AZD2281 at 2 μM significantly inhibits cell survival in all 4 cell lines, whereas ABT-888 and BSI-201 did not induce cell death at 2 μM (Fig. 1b). We also evaluated the effects of AZD2281 at lower concentrations in the BRCA mutant breast cancer cell lines, where it had a significant dose-dependent growth inhibition effect (Fig. 1c).

### BRCA1 or BRCA2 downregulation in BRCA wild-type breast cancer cell lines induces growth inhibition in response to AZD2281 treatment

**Figure 2:**
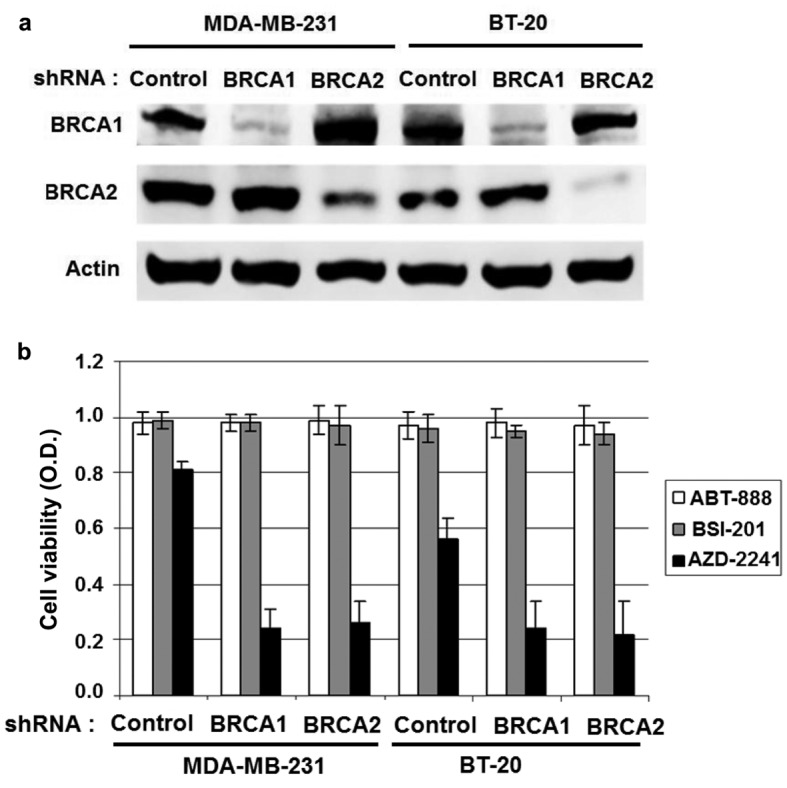
BRCA1 or BRCA2 downregulation in BRCA wild-type TNBC cell lines enhances growth inhibition in response to AZD2281 treatment (day 4). (a) The *BRCA1* and *BRCA2* wild-type TNBC cell lines (MDA-MB-231 and BT-20) were stably transduced with lentiviral-BRCA1, BRCA2, or control shRNA and western blot analysis was performed to demonstrate downregulation of BRCA1 or BRCA2 protein expression levels. (b) Control and BRCA1- or BRCA2-knockdown TNBC cell lines were subjected to AZD2281 treatment. Cell survival was was measured 4 days after the treatments using WST-1 assay.

To determine the effect of BRCA1 or BRCA2 in response to AZD2281 treatment, the BRCA wild-type MDA-MB-231 and BT-20 cells were stably transfected with BRCA1, BRCA2, or control lentiviral shRNA. BRCA1 or BRCA2 downregulation was demonstrated by western blot analysis (Fig. 2a). The 3 different PARP inhibitors used as single-agent treatments and growth rates were measured with the WST-1 assay. AZD2281 induced significantly superior growth inhibition compared with other PARP inhibitors, such as ABT-888 and BSI-201 in BRCA1- or BRCA2-knockdown cells than in control cells, indicating that the growth inhibition effect of AZD2281 is dependent on BRCA deficiency (Fig. 2b).

### AZD2281 induces autophagy in BRCA1 or BRCA2 mutant breast cancer cell lines

**Figure 3:**
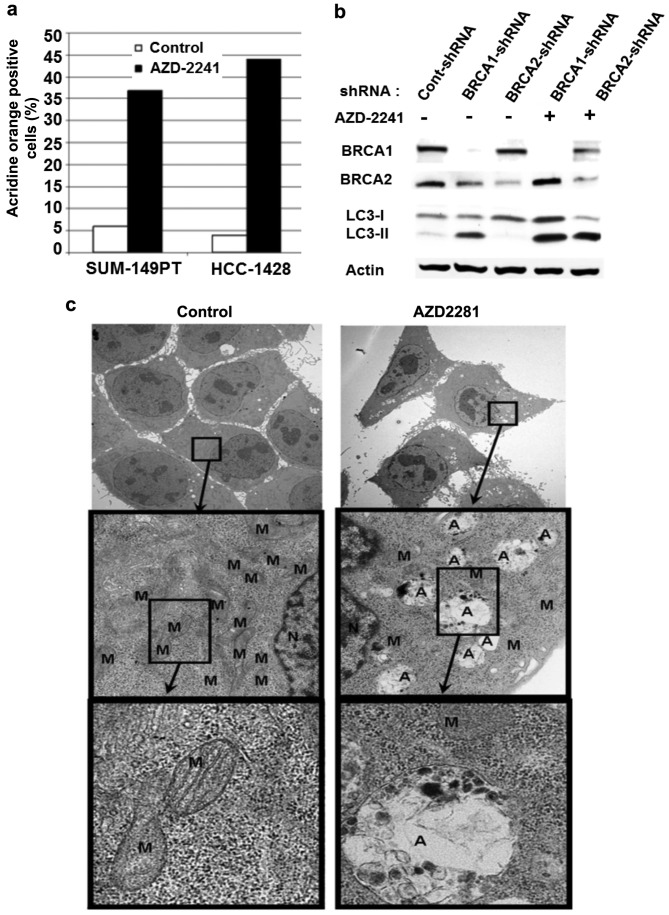
AZD2281 treatment induces autophagy in *BRCA1* or *BRCA2* mutant or *BRCA1* and *BRCA2* knockdown TNBC lines. (a) *BRCA1* mutant (SUM-149PT) and *BRCA2* mutant (HCC-1428) breast cancer cell lines were treated with AZD2281 (2 μM) and stained with acridine orange and acridine orange-positive cells were quantified by flow cytometry to demonstrate formation of acidic vacuoles (Akar 2008). (b) BRCA1 and BRCA2 protein expression was knocked down in the BRCA wild-type MDA-MB-231 cells by using lentiviral BRCA-shRNA, BRCA2-shRNA control-shRNA expression vector. Western blot analysis was performed after AZD2281 (2 μM) to demonstrate expression of BRCA1, BRCA2, and LC3-II protein, a marker for autophagy. (c) The BRCA-mutated HCC-1428 cells were treated with AZD2281 (2 μM) or vehicle control and analyzed by transmission electron microscopy (TEM). After 1 day of treatment, cells were fixed for TEM micrographs to show cellular organelles and ultrastructures. Increased autopghagosome formation (indicated by letter A, autophagosome) and reduction of number of mitochondria (labeled M, mitochondrium) were observed in AZD2281-treated cells.

Autophagy is lysosomal degradation pathway characterized by an increase in the number of autophagosomes that surround organelles such as mitochondria, Golgi complexes, polyribosomes, and the endoplasmic reticulum. Subsequently, autophagosomes merge with lysosomes and digest damaged organelles into amino acids to provide a new supply under stressful conditions to protect the cells (22–24). Although activation of autophagy is aimed at overcoming stressful situations, autophagy induction may lead to cell death (25). To determine effects of the most potent PARP inhibitor we investigated whether AZD2281 induces autophagy in BRCA mutant breast cancer cell lines. To this end we treated *BRCA1* mutant (SUM-149PT) and *BRCA2* mutant (HCC-1428) breast cancer cells with 2 μM AZD2281 for 1 day and stained them with acridine orange. Acridine orange positive cells were counted using flow cytometry. AZD2281 induced significant autophagy (37 and 44%) in *BRCA1* mutant SUM-149PT and *BRCA2* mutant HCC-1428 breast cancer breast cancers, respectively, in 24 of treatment (Fig. 3a). We observed the same phenomenon by AZD2281 in BRCA wild-type breast cancer cell line MDA-MB-231 with BRCA1 or BRCA2 downregulation. The knockdown of BRCA1 by lenti-based stable shRNA in BRCA wild-type breast cancer cell line MDA-MB-231 demostrated induction of autophagy as indicated by the expression of LC3-II, an autophagy marker (Fig. 3b). AZD2281 treatment further enhanced LC3-II expression in BRCA1- or BRCA2-knockdown cells (Fig. 3b).

To further demonstrate the induction of autophagy we also investigated ultrastructure by transmission electron microscopy (TEM) before and after AZD2281 treatment. TEM images clearly demonstrated that AZD2281 induces autophagy, which results in mitochondrial degradation. AZD2281-treated cells had fewer mitochondria and more autophagosomes compared with untreated cells (Fig. 3c).

### Inhibition of autophagy results in partial inhibition of AZD2281-induced apoptosis

**Figure 4:**
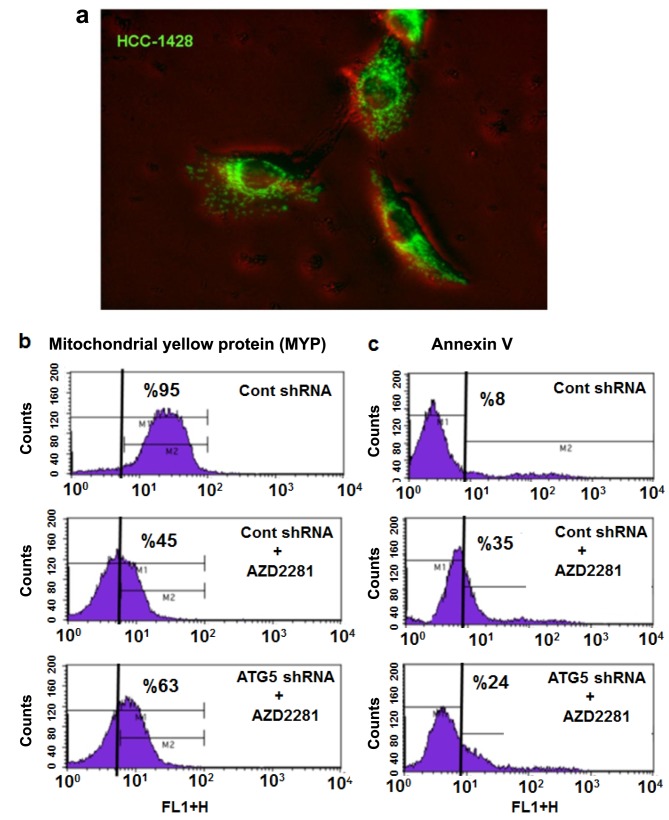
AZD2281 treatment leads to reduction in number of mitochondria through induction of autophagy. (a) The HCC-1428 cells were stably transduced with lentiviral vector expressing mitochondria yellow protein (mYFP) and imaged by fluorescence microscopy. (b) The *BRCA2* mutant cell line HCC-1428. mYFP was stably transfected with either control shRNA or ATG5 shRNA and treated with AZD2281 or vehicle control. Mitochondria were measured by flow cytometry. (c) Apoptosis was assessed by using Annexin V staining and flow cytometry analysis.

To investigate the roles of autophagy and mitochondrial degradation under AZD2281 treatment, we stably transfected HCC-1428 cells with mYFP using lentiviral vector. Fluorescence microscope images clearly demonstrated the presence of mYFP in the mitochondrial compartment of HCC-1428-mYFP cells (Fig. 4a). HCC-1428-mYFP was treated with AZD2281, and mitochondrial fluorescein was measured by flow cytometry; untreated cells were used as a control. AZD2281 induced significant mitochondrial degradation (~45%) (Fig. 4b), which was also shown in the TEM images. Next, we inhibited autophagy by knocking down the key autophagosome structural protein ATG5 using lentiviral shRNA vector in HCC-1428-mYFP cells. Mitochondrial degradation was markedly rescued in ATG5-knockdown HCC-1428.mYFP-shATG5 cells compared with HCC-1428-mYFP-sh-control cells under AZD2281 treatment (Fig. 4b). Inhibition of autophagy by knocking down ATG5 also partially inhibited AZD2281-induced apoptosis (Fig. 4c), suggesting that autophagy contributes to AZD2281-induced cell death in BRCA mutated breast cancer cells.

## Discussion

In this study, we show for the first time that a PARP inhibitor as a single agent induces significant autophagy/mitophagy in *BRCA* mutant cell lines. In addition, we demonstrated that AZD2281 induces growth inhibition in BRCA wild-type breast cancer cell lines with *BRCA1* allelic loss, indicating that breast cancer patients with *BRCA1* allelic loss may benefit from PARP inhibitors.

**Figure 5:**
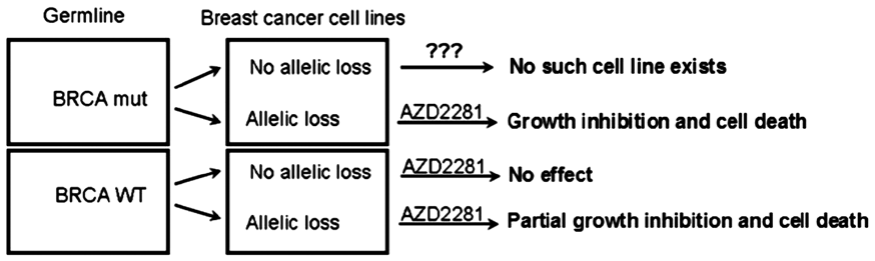
Effect of AZD2281 treatment on breast cancer cell lines based on their *BRCA1* and *BRCA2* mutation and BRCA allelic loss status.

Previously, AZD2281 was evaluated in a genetically engineered mouse model of BRCA1 breast cancer (26). Treatment of tumor-bearing mice with AZD2281 inhibits tumor growth and prolonged survival. Combination treatment with AZD2281 plus cisplatin or carboplatin increased recurrence-free survival and overall survival (26). AZD2281 has also been used as a single agent in clinical trials in breast and ovarian cancer patients with BRCA mutations (19,20). In this study, we evaluated the effects of 3 different PARP inhibitors, ABT-888, BSI-201 and AZD228, in BRCA mutant breast cancer cell lines as single agents without DNA damaging agents; such a study has not been performed previously. BRCA mutations in breast cancer cell lines were not well described until 2006, when Elstrodt *et al*, reported a detailed *BRCA1* mutation analysis of 41 breast cancer cell lines (5). Before the report was published, only one of the 41 cell lines was known to have *BRCA1* mutation. Elstrodt *et al*, identified *BRCA1* mutations in three cell lines that had not been described as *BRCA1* mutant before. They also found that 28 (68%) of the 41 cell lines had *BRCA1* allelic loss (5). On the basis of these results, we evaluated PARP inhibitors as single-agent therapy in 14 breast cancer cell lines: 4 BRCA mutant lines with *BRCA1* allelic loss, 9 BRCA wild-type lines with *BRCA1* allelic loss, and 1 BRCA wild-type line without *BRCA1* allelic loss. Our data clearly demonstrated that BRCA mutant breast cancer cell lines with BRCA allelic loss were highly sensitive to AZD2281 as monotherapy (Fig. 5). Unfortunately, no cell line exists with BRCA mutation and without BRCA allelic loss; such cells may be resistant to PARP inhibitors because of a functional BRCA allele. When we investigated whether BRCA allelic loss results in sensitivity to PARP inhibitors in BRCA wild-type cell lines, we found significant growth inhibition, but not cell death, such as that seen in BRCA mutant cell lines.

Autophagy is lysosomal degradation pathway that is induced as a protective and prosurvival pathway against nuclear DNA damage and metabolic and therapeutic stress, if excessive this process can also lead to cell death in breast and other cancers (22–25,29,30). To the best of our knowledge, our study is the first to show that AZD2281 induces complete cell death (95–99%) and autophagy, which targets mitochondria. Our findings indicate that autophagy is involved in cell death mechanism as AZD2281-induced apoptosis was reversed by genetic inhibition of autophagy. Here, we speculate that AZD2281 not only induces nuclear DNA damage but may also induce elimination of mitochondria by autophagy by a process called mitophagy and may contribute to the cell death process (28). Although the clinical implications of this finding are not yet known, we speculate that autophagy could serve as a predictive marker for PARP inhibition therapy. Furthermore, our study points out that BRCA wild-type cells with BRCA allelic loss may be more sensitive to PARP inhibitors than are those without BRCA allelic loss. This observation may potentially explain why differential response rates are being observed in clinical trials, even in homogeneous cohorts of germline BRCA mutation carriers. For example, the reported response rate is ~40% for AZD2281 and ~37.5% for ABT-888 (in combination with temozolamide), indicating that almost half of the patients with germline BRCA mutations are not responsive to these agents (20,27). Therefore, the results of our current study might shed further light on the molecular subclassifications of BRCA-related breast cancers and ultimately lead to a better characterization of the molecular tumor type that would benefit from PARP inhibitors.
